# Challenges of Applying Continuing Education in Tehran Hospital Practice as Viewed By Nurses

**Published:** 2015-04

**Authors:** Laleh Khosravi, Nahid Dehghan Nayeri, Tahmineh Salehi, Anoshirvan Kazemnejad

**Affiliations:** 1 Department of Nursing Management, School of Nursing and Midwifery, Tehran University of Medical Sciences Tehran, Iran;; 2 Department of Nursing Management, Nursing and Midwifery Care Research Center, School of Nursing and Midwifery, Tehran University of Medical Sciences, Tehran, Iran;; 3 Department of Biostatistics, Tarbiat Modares University, Tehran, Iran

**Keywords:** Continuing Education, Nursing, Professional Nurses

## Abstract

**Background:**

Although many planners of professional continuing education programs believe that this type of education positively affects the nurses’ performance, the results obtained by conducted research do not confirm such a perspective. In fact, inadequate application of these trainings in clinical practice is among the most challenging areas in nursing practices. Hence, this study was conducted to describe the challenges nurses encounter in order to apply what they have learned during continuing education programs in clinical settings of TUMS hospitals.

**Methods:**

This cross-sectional study was conducted on 400 medical-surgical nurses who worked in the hospitals of Tehran University of Medical sciences. For sampling, after listing all the general hospitals, their wards were selected in proportion to hospital. Nurses filled out a questionnaire about factors affecting the application of continuing education. The questionnaire contained 43 items and the dimensions were supportive-organizational, individual, professional, and educational program design factors. The analysis was carried out using parametric and non-parametric method using SPSS 16 package.

**Results:**

The results showed while 48.5% and 53.8% of nurses mentioned organizational and professional factors, respectively as the most inhibiting factors; only 2.25% of the nurses believed that organizational factors are facilitating.

**Conclusion:**

The results obtained in this study are important regarding the fact that organizational and professional factors have a key role in applying or lack of application of learned materials. Thus, hospital authorities as well as nursing managers can provide the necessary condition in application of continuing education through promotion of facilitating factors and eliminating the hindering ones.

## Introduction

Updating nurses’ medical knowledge and skills is one of the most important nursing matters, besides applying new skills in providing nursing care is a necessity.^1^ Also, this type of education should be purposefully planned^2^ and efficiently applied in clinical practice in order to have its effect. Moreover, despite numerous educational programs the gap between scientific developments and nurses’ capabilities is still significant.^3^ The results of a study indicated that participation in continuing education programs has not caused any changes in nurses’ performance.^4^

Furthermore, other researchers claim that professional continuing  education is effective when nurses apply a suggested behavior or performance in their clinical practice; however, the results of studies about the effect of continuing education on  nursing practice does not confirm such a claim.^5^ The basis of adults’ continuing education is this assumption that development of knowledge by itself does not guarantee the use of that knowledge or any behavioral changes.^6^ Authors mention that continuing education mainly focuses on cognitive aspects of nursing performance and is unable to make any advancement in critical thinking and its reflection on operational skills and the new knowledge isn’t necessarily put into practice.^7^

Iranian researchers believe that although the launch of continuing education programs goes back to a relatively long time ago and, therefore, it is expected that they would improve the quality of nursing care by now; unfortunately, the positive effects of such education on nurses’ professional performance and, as a result, improvement of nursing care quality have not been noteworthy.^8^ Hence, application of learning materials and transferring them into the clinical practice must be earnestly looked into. A number of studies investigated both knowledge level and behavioral changes of the staff. The results indicated that staffs’ knowledge has increased significantly by the end of educational courses while their behaviors were exactly the same.^9^ This finding implies that there are a number of factors, some problematic and some facilitating, affecting the process of transferring knowledge into practice. Therefore, based on the nurses’ experience, the authors of this study decided to investigate the aforementioned factors in application of continuing education programs in clinical practice. This study is the one of the first studies that examines the barriers of and facilitators to applying what is learned from continuing education programs. In addition, the questionnaire of this study has been made by a mixed method approach and all of the validation stages were completed.

Through recognizing these factors, the educational experts and nursing managers can apply relative strategies in order to enhance the efficacy of educational programs and provide a suitable environment to apply the learned materials. This process, in turn, leads to quality improvement of patients’ care and efficacy of the educational programs. 

## Materials and Methods

This is a descriptive cross-sectional study based on the nurses’ experience. The research samples include 400 nurses working in hospitals of Tehran University of Medical Sciences. Nurses with at least 6 months of experiences who had participated at least twice in continuing education programs were randomly selected by cluster method. This study is a MSN Thesis that was conducted in 2012 and 2013.

The study sample size was determined with a=0.05, d=0.05 and P=0.5. We surveyed the studies  by relying on the previous studies conducted by Stolee et al.^10^ However, not only in this study but also in other studies, detailed statistics about the impact of factors had not been provided; thus, we took the P=0.5 for formula to achieve maximum sample size. The formula for estimating the sample size used in the study was as follows.

$$N=\frac{z_{1-a/2}(P\left[1-P\right])}{d^{2}}$$

The sample size estimated was 385. However, finally we collected 400 questionnaires. 

For sampling process, first all the general hospitals in Tehran were listed. Then, a list was prepared from all the medical-surgical wards. It was assumed that on average 15 nurses work in each ward. In whole, 11 hospitals were entered into the sampling. Therefore, considering the total estimated number of samples (nurses), the number of wards was calculated as follows: 425/15=29. These wards were selected as a proportion of all the wards in each hospital. A number was given to every ward. Then, 29 numbers were selected randomly by using a table of random number. 

The research proposal was approved by the ethics committee of Tehran University of Medical Sciences and the authorities of the university and hospitals issued permission for the researchers to enter the research environment. Then, all the staff from different working shifts in the hospitals that had the conditions and were willing to take part in the study were given the research questionnaires. Researchers gathered the questionnaires after they were filled out by the staff. Returning the questionnaires was considered as consent to participate in the study. 

In order to collect information, a two-part questionnaire was used. Part one was on demographic characteristics and the second part was about facilitating and problematic factors in application of continuing education in clinical practice. The questionnaire of factors contributing to application of continuing education was designed based on the results of a qualitative study conducted by Dehghan Nayeri and Khosravi,^11^ in which all the instrumentation steps have been completed. After developing the items and defining the measuring standards, the researchers revised all the items several times. They also took into account the recommendations and remarks of a panel consisting of 12 nursing experts in management and continuing education. The panel members offered their comments in written form about linguistic structures and use of appropriate expressions in the course context. After employing the experts’ comments in the first step and modifying the items, Content Validity Ratio (CVR) was used to justify the presence of each item, and Content Validity Index (CVI) to evaluate the clarity of items and their relevance to the study objectives; then, based on these evaluations some items were removed or modified.

In order to examine face validity and usability of the items, they were tested by 15 nurses and based on their comments some changes were applied. Validity of the items was checked based on participants’ opinion about items difficulty, ambiguity, and relevance. Moreover, the tool was filled by 30 qualified nurses who were not among the study samples. The calculated α Cronbach’s ratio (0.9325) also indicated the reliability of the tool. After these steps, the tool was factor analyzed by 380 samples and at the end 43 items remained in areas including supportive-organizational factors (20 items), individual (8 items), professional (9 items), and educational program design (6 items). 

Items such as “climate of welcoming new knowledge and enthusiasm to accept change, existence of supportive rules for applying CME, authorities’ vision and attention to the role of education, adequate medical and authorities support, attitude of providing high quality care, considering applying of education as a privilege for annual evaluation, enough facilities and financial incentives for applying what learned” formed supportive-organizational factors. 

Some of the items for individual factors included scientific character of the person, motivation and sufficient liability for learning, changing and applying what is learned, commitment, consciousness, and professional interest.

Professional factors consisted of items, such as lack of advances in duties in accordance with the development of science and technology, having autonomy for applying what is learned, experienced personnel’s resistance to change, and routine-orientation. 

Some items of educational program were fitness of CME with educational needs of the staff and ward, appropriate quality of educational content, and updated education content. 

The items were arranged in Likert scale from high to low; all positive items were given scores of 5 (high) to 1 (low), respectively. Scoring process for negative items was done inversely. Thus, the ranges of dimensions were respectively individual factors (8-40), professional (9-45), supportive-organizational factors (20-100), and educational program design (6-30). The higher scores indicated that more facilitating factors existed on that dimension and lower scores showed that more hindering factors exist.

Finally, scores higher than 75% were indicated as facilitating factors, and those lower than 50% as problematic and delaying factors. The gathered information was analyzed using SPSS, version 16 by Pearson, Chi-square, and regression tests. Wherever assumptions were met, the parametric statistics, such as Pearson correlation coefficient was used. If the data were at the nominal or categorical level, non-parametric statistics such as chi-square test was used. 

## Results

Analysis of demographic characteristics indicated that the nurses’ age varied from 22 to 50 (M=33.01±6.09). Also, 82.5% of the nurses were female, 61.8% were married, and 71.25% had 1 or 2 children. The majority of participants (85.8%) were workings nurses and 92.5% of them had bachelor’s degrees. About half of the participants (49.88%) were working in circulating shifts and 55% of them had clinical work experience of less than 10 years. In addition, 55% of the participants were working as permanent employees and the majority of them (76.08%) had no managerial work experience. 

The results obtained from the part regarding facilitating and problematic factors in continuing educational programs indicated that nurses had, at best, experienced the facilitating aspect of all the factors in less than 20% of the time. Most facilitating factor in their experiences was the individual factor (16.25%).

Just a few of nurses (10%) found the role of professional factors facilitating in application of continuing education while more than half of them (53.8%) had specified professional factors as challenging. 

Furthermore, only 2.25% of the participants found organizational factors facilitating whereas nearly half of them (48.5%) found the factors delaying and problematic. 7.2% had recognized the design of educational programs as a facilitating factor; while for about half of them (51.25%) these factors were problematic (Table 1).

**Table 1:** Distribution of nurses’ experiences of facilitating and delaying factors about applying of continuing education program

**Factors **	**Score **	**Number **	**Percent**
Individual	8-16	28	7
	17-24	90	22.5
	25-32	217	54.25
	33-40	65	16.25
	M=27.35±6.1		
Professional	9-18	24	6
	19-27	191	47.8
	28-36	145	36.2
	37-45	40	10
	M=26.24±5.98		
Organizational	20-40	56	14
	41-60	194	48.5
	61-80	141	35.25
	81-100	9	2.25
	M=67±12.5		
Education program	6-12	14	3.5
	13-18	152	38
	19-24	205	51.25
	25-30	29	7.25
	Total	400	100
	M=19.33±3.68		

The relationship among different dimensions is shown in the Table 2.

**Table 2:** Relationships among different dimensions of factors

	**Organization factors**	**Individual factors**	**Professional factors**	**Program education **
Organization factors	1	0.630**	0.285**	0.604**
Individual factors	0.630**	1	0.266**	0.550**
Professional factors	0.285**	0.266**	1	0.237**
Program education factors	0.604**	0.550**	0.237**	1

^**^P<0.001

Analyzing the relationship between demographic characteristics and the above-mentioned factors revealed that there is a significant link among professional factors, age (r=+0.139, P=0.007), work experience (r=0.124, P=0.015), and managerial experiences (r=0.122, P=0.016).

In addition, the results of Chi-square test showed that there was a relationship between the participants’ gender, educational programs design (P=0.034), and professional factors (P=0.021). There is also a noteworthy relationship between the nurses’ employment status and individual factors.

Furthermore, the results of Fisher statistical test revealed that there was a significant relationship between marital status and factors regarding educational programs (P=0.040). There was also a connection between the nurses’ working shifts and organizational factors (P=0.006).

After examining the relationships between demographic variables and dimensions, regression analysis was conducted for testing the variance accounted for by the set of independent variables. We entered any relationship above with the p value >0.2 into the model. There was not any significant result in the regression model of organizational and individual factors. The regression model for professional factors is shown in Table 3. Sex, Position and Work Shift are the predictors of professional factor in this model.

**Table 3:** Regression model of Professional Factors

**Model **	**Unstandardized Coefficients**		**Standardized Coefficients**	**t**	**Sig.**
	**B**	**Std. Error**	**Beta**		
(Constant)	29.612	3.665		8.079	0.000
Age	-0.159	0.123	-0.164	-1.294	0.197
Clinical experience	0.055	0.130	0.054	0.419	0.676
Management Post	-1.292	0.714	-0.131	-1.810	0.071
Sex	2.209	0.861	0.142	2.566	0.011
Marriage Status	-1.154	0.656	-0.094	-1.760	0.079
Position	1.604	0.761	0.146	2.107	0.036
Shift	0.445	0.204	0.127	2.180	0.030
Employment Status	-0.595	0.368	-0.104	-1.618	0.107

B=Unstandardized Regression Coefficient

The regression model of educational programs design showed that only the participants’ degree can be a predictor of the variable (Table 4).

**Table 4:** Regression model of Educational Program Design Factors

**Model**		**Unstandardized Coefficients**		**Standardized Coefficients**	**t**	**Sig.**
		**B**	**Std. Error**	**Beta**		
1	(Constant)	16.929	1.171		14.452	0.000
	Clinical experience	-0.008	0.041	-0.014	-0.207	0.836
	Management experiment	0.232	0.333	0.041	0.697	0.486
	Sex	0.039	0.521	0.004	0.075	0.940
	Marriage Status	0.713	0.393	0.095	1.812	0.071
	Degree	1.346	0.485	0.147	2.777	0.006
	Employment Status	-0.216	0.220	-0.061	-0.980	0.328

B=Unstandardized Regression coefficient

## Discussion

According to this study, the nurses’ experience of applying continuing education programs in clinical practiceimplies that the facilitating role of all the factors – individual, organizational, professional, and educational programs design – is very insignificant; nevertheless, the role of individual factors was the most effective ones in nurses’ opinion. 16.25% of them believed that individual factors were facilitating while 29.5% of them considered them to be problematic and delaying. 

Individual factors, such as individuals’ scientific level, work experience and motivation to apply continuing education in nursing, have been widely emphasized. Other researchers also pointed out that staff personality influences their organizational behaviors.^12^ Participants of the qualitative research conducted by Nayeri & Khosravi frequently emphasized the importance of individual characteristics and differences as influencing factors in the overall process of application of continuing education programs{Nayeri, 2013 53 /id}. Also, it was found that individuals who are known as knowledge sources in the organization are a significant factor in application of the new knowledge in practice by the nurses.^13^ Also, the results of Blair’s research showed that the nurses with higher educational degrees – educational level was among the investigated factors of the present study – have higher skills in finding new materials, literature review, and application of evidence-based results.^14^

One of the most important findings of this research showed that as nurses’ age increased, they become less motivated to apply newly learned knowledge in their practice. The other researchers have also reached similar results; for example, Baumann found that willingness to apply  new learned materials and nurses’ creativity are inversely related to their age.^15^

According to this study, another factor that causes problem in application of continuing education programs is lack of job satisfaction and motivation. Even with the mentioned points, commitment to work had relatively more facilitating effect on application of learned materials. Other researchers also indicated that motivation is a key factor in effectiveness of continuing education programs. They also found that other important factors in efficacy of continuing education programs are responsibility, commitment, and the type of employers’ evaluation system in transferring new learned materials to clinical practice.^10^

The current study results showed that about half of the participants considered professional factors delaying and problematic. Moreover, other researchers have pointed out that professional factors, such as authority, independence and power are effective means in application of theoretical material in clinical practice. In this study, participating nurses considered professional factors such as lack of enough independence in the job  as a delaying factor in application of their new learned materials. In another research conducted in Iran, it was reported that only 12.2% of the nurses have assessed their working conditions as productive.^16^ In addition, Wilson mentions that having a sense of authority at work improves the nurses’ independence and their team work skills. It also offers more freedom to them to apply their new learned materials in order to fill the current gap between theory and practice.^17^ Researchers also believe that factors related to job and its development, such as application of learned materials and new findings, influence the nursing staff.^18^

Professional factors which are negatively affecting the application of continuing education are the routine-centric approach in clinical practice and wrong motivations to participate in the programs such as getting a promotion and consequently its financial advantages. Researchers think that routine centrism is a complication in nurses’ professional development.^19^ The result of another study  entitled “Pathology of continuing education programs in Iranian Medical Society” showed that the majority of  the participants (75%) believed that if continuing education was not followed by financial/promotional benefits, the number of participants in these programs would drastically decrease.^20^

Besides, the participants found factors related to designing and executing the educational courses challenging. Xiao believes that educational programs still have to deal with some critical issues.^21^ However, most researchers believe that educational programs must be designed and presented according to the learners’ needs^22^ and there must be a proper cooperation between organizers of educational programs and clinical nurses in order to achieve the highest efficiency.^23^ As to educational factors, most nurses believe that e-learning programs are ineffective. Lynn et al found that the best method available for offering new materials is face-to-face education.^24^ Nevertheless, some researchers believe that the traditional programs are time-consuming, expensive, and demanding, while e-learning courses are available round-the-clock, there is no need to travel in order to attend the classes and it saves about 25% to 30% of the participants’ time.^25^ However, the participants of study talked about some problems in the e-learning system such as the offered materials, teaching method, and inappropriate evaluation system. While most of participants in this study considered the content of educational programs as ineffective, some researchers believe that high quality continuing learning programs can be offered by using educational lectures, conferences, booklets, and video conferencing if they are to take the learners’ needs into accountand learners continuously evaluate the offered materials.^26^

Results of this study regarding organizational factors demonstrate that only 2.25% of the participants consider the role of organizational factors in application of the learned materials as facilitating. Estabrooks et al. also believe that nurses should not ignore organizational and social factors and the policies that could affect application of learned materials.^27^ Other researchers also emphasize the importance of organizational factors, especially in application of new findings and learned materials.^28^ They consider the lack of financial and organizational support as a hindering factor.^29^

In this section, the most challenging factors in the nurses’ point of view are lack of balance between workload and the number of personnel, insufficient nursing budget, and inadequate support offered by the managers. Others mention that although nurses expect to be given opportunities in order to put their theoretical knowledge into practice, Iranian hospitals do not offer such opportunities, due a number of issues such as insufficient human resources.^30^ Stolee et al. emphasized the necessity of sufficient financial resources as well as managers’ support on the way to effective application of learned materials.^10^ In another study, the participants complained about the managers’ discouraging attitude and their lack of interest in supporting continuing education programs.^31^ All of the afore-mentioned studies confirm the result of the present study.

*Limitation and Suggestion*

This study was conducted in public hospitals in Tehran. Thus, the results of the study may not be generalizable to the hospitals in different cities with different organizational structure, climate and culture. Therefore, it is recommended that further studies should be conducted in other cities. Since the organizational structure and personnel composition of the private hospitals is different, it is suggested that a study should be conducted in private hospitals. 

## Conclusion

This study was conducted to find out the “Challenges of applying continuing education in clinical practice”. According to the results, nurses have estimated the positive role of all factors quite insignificant in application of continuing education programs. In this regard, they considered the organizational factors to be the most challenging. Hence, by providing professional and moral support, the authorities, and hospital and nursing managers can make a productive atmosphere available for nurses’ professional improvements. 
